# Locoregional Approaches in Cholangiocarcinoma Treatment

**DOI:** 10.3390/cancers14235853

**Published:** 2022-11-28

**Authors:** Alexander E. Hare, Mina S. Makary

**Affiliations:** Division of Vascular and Interventional Radiology, Department of Radiology, The Ohio State University Wexner Medical Center, Columbus, OH 43210, USA

**Keywords:** cholangiocarcinoma, transarterial chemoembolization, transarterial embolization, ablation

## Abstract

**Simple Summary:**

Our current ability to treat cholangiocarcinoma is limited. Surgery to remove the tumor is only possible in a small proportion of cases of the disease due to the tumor’s location or spread to other sites in the body. When surgery is not an option, locoregional therapies that target their treatment to a small region have the potential to slow progression, decrease the size of the tumors, increase overall survival, and in some cases, allow for cure. This review will discuss what locoregional therapies exist, their role is in treatment of cholangiocarcinoma, and their impact on patients.

**Abstract:**

Cholangiocarcinoma (CCA) is a rare hepatic malignant tumor with poor prognosis due to late detection and anatomic factors limiting the applicability of surgical resection. Without surgical resection, palliation is the most common approach. In non-surgical cases contained within the liver, locoregional therapies provide the best chance for increased survival and disease control. The most common methods, transarterial chemoembolization and transarterial radioembolization, target tumors by embolizing their blood supply and limiting the tumor’s ability to metabolize. Other treatments induce direct damage via thermal ablation to tumor tissue to mediate their anti-tumor efficacy. Recent studies have begun to explore roles for these therapies outside their previous role of palliation. This review will outline the mechanisms of each of these treatments, along with their effects on overall survival, while comparing these to non-locoregional therapies.

## 1. Introduction

Cholangiocarcinoma (CCA) is an epithelial cell malignancy typified by features of cholangiocytes that can occur at multiple locations throughout the biliary tree. It is the second most common hepatic malignant tumor in the West, with incidence of approximately 2.1 cases per 100,000, and incidence continues to rise [1]. CCA is much more common in Asia, with incidence as high as 113 per 100,000 [2]. However, CCA only makes up 3% of gastrointestinal malignancies and 15% of primary liver cancers. This is contrasted with the significantly more common hepatocellular carcinoma (HCC), a cancer of hepatocytes, and located in the same region [3]. CCA typically has a poor prognosis, which can be ameliorated with early detection. In terms of anatomic location, CCAs are defined as either intrahepatic, which comprises up to 10% of CCA, or extrahepatic [4,5]. Extrahepatic CCA is divided into distal (30%) or perihilar (60%) categories. The location of a tumor is particularly important because, historically, the primary curative treatment for CCA is surgical resection. In addition to surgical resection, liver transplantation has been used for perihilar CCA that is otherwise unresectable [6,7]. Survival of patients without treatment is poor, with a median overall survival time of 3.9 months, ranging from 0.2–67.1 months [8]. Typically, CCA present as aggressive tumors, with most patients presenting with their disease in an advanced stage [9]. Earlier diagnosis is hampered by the asymptomatic nature of early-stage disease and the fact that CCA’s clinical presentation broadly depends on the tumor’s location, stage, and growth. This difficulty with early diagnosis directly leads to detection of disease in an unresectable state, which necessitates the use of locoregional methods for treatment. CCA is associated with parasitic infection, cirrhosis, hepatolithiasis, biliary-duct cysts, inflammatory bowel disease, hepatitis B and C viruses, and primary sclerosing cholangitis (PSC), among others [10].

### 1.1. Intrahepatic Cholangiocarcinoma

Using the Liver Cancer Study Group of Japan (LCSGJ) classification, intrahepatic CCA (iCCA) grows in three primary patterns: mass-forming (within the liver parenchyma, most common), periductal-infiltrating (along and within bile duct), and intraductal (contained within the lumen of the bile duct) [11]. The mass-forming and periductal-infiltrating iCCA have a poorer prognosis and higher rates of recurrence [12,13]. The presentation of iCCA is nonspecific, including abdominal pain, weight loss, night sweats, and gastrointestinal disturbances [14]. Jaundice is uncommon, found in approximately 20% of patients. All these non-specific findings lead to difficulty in detecting iCCA, and most that are detected are incidental. Detecting iCCA is performed using CT and MRI. Tumor marker measurements of CA19-9 and CA242 have been utilized in distinguishing iCCA from hepatocellular carcinoma (HCC), but have limited utility in detection compared to monitoring for recurrence [15]. However, no data suggest that definitive diagnosis can be made prior to biopsy [16].

### 1.2. Extrahepatic Cholangiocarcinoma

Extrahepatic cholangiocarcinoma (eCCA) is divided into perihilar CCA (pCCA) and distal CCA (dCCA). pCCA is categorized by the UICC and WHO, who use differing definitions. The Bismuth-Corlette classification of pCCA stratifies by ductal infiltration [17]. In the UICC/AJCC classification, the threshold between iCCA and pCCA is the second-order bile ducts and between dCCA by the insertion of the cystic duct, whereas the WHO system defines it as arising from the intrahepatic bile duct epithelium [18].

Due to their anatomic location, eCCA presents with biliary symptoms such as painless jaundice in 90% of patients, and cholangitis in 10% of patients [19]. Due to this apparent presentation, eCCA can be diagnosed earlier and in an earlier stage than iCCA, which is associated with a more positive prognosis. Beyond biliary symptoms, eCCA can present with symptoms typically associated with malignant disease, such as anorexia, fatigue, and weight loss, in 56% of patients [20]. Laboratory abnormalities such as elevated liver function tests and alkaline phosphatase can support diagnosis of eCCA vs. iCCA due to the disruption of the biliary tree. This can present on physical exam with a palpable enlargement of a single hepatic lobe [21]. Due to the presence of painless jaundice, diagnostic workup of eCCA often begins with ultrasound, which is neither specific nor sensitive for eCCA. However, cross-sectional imaging, typically contrast CT or MRI/MRCP, is the next diagnostic test and is the most important test for detection [22]. PET/PET-CT is less sensitive for eCCA (approximately 60%) compared to iCCA (greater than 90%) and cannot be relied upon for detection of iCCA, though it has value in the staging of both iCCA and eCCA [23]. Cholangiography also has significant benefit in visualizing the biliary tree and preoperative planning [24,25].

### 1.3. Non-Locoregional Therapies in Cholangiocarcinoma

Collectively, CCA has poor prognosis and poor overall survival. One study found that dCCA had 1-, 3-, and 5- year overall survival of 46%, 18%, and 11% [26]. This study included patients treated with resection and palliative chemotherapy for metastatic disease. Another study compared HCC, gallbladder cancer, and CCA, with the finding that CCA had median survival time of 180 days and 600 days if patients receiving best supportive care were excluded [27]. The study analyzed patients treated between 2009 and 2016 and noted significantly increased (*p* < 0.011) survival rates over time. In addition to later diagnosis of CCA, overall survival rates in CCA are negatively affected by the high rates of recurrence [28]. More than half of patients with pCCA experienced recurrence, even when microscopic investigation of the margins was negative, called R0 resection [29]. One study, which found an overall survival of 40 months, found a recurrence rate of 76% in patients at eight years, including a 28% recurrence rate in patients who had already experienced a 5-year recurrence-free period [30]. This highlights the difficulty in achieving durable remission in CCA. Adjuvant chemotherapy and radiotherapy were found to be associated with worse 5-year survival (*p* < 0.001), due largely to their association with node- and margin-positive disease. Poor prognostic factors for CCA include increased age, greater tumor invasion, higher lymph node ratio, poor differentiation, and resection with positive margins [26].

Thus far, surgical resection is the first line choice for curative intent in patients in any type of CCA [31,32]. As surgical resection is the only curative option for CCA with significant efficacy, resection is indicated in any case in which it is possible, given the anatomical and clinical picture. Resectability can depend on anatomic location. Generally, the purpose of surgical resection is to remove the involved bile ducts and the portal lymph nodes draining the involved region, focusing on a surgical outcome with negative margins. Due to location in iCCA and pCCA, lobar removal of the liver is often required. Due to this loss of liver tissue, the volume of functional liver post-surgery needs to be considered to promote the patient’s functional status. Generally, resection will require at least 25% of healthy preoperative liver to survive, with higher thresholds for unhealthy livers. Both locoregional and distance metastases can occur. One study showed disease recurrence in 53% of patients with pCCA, even after R0 resection [33]. In that study, the majority (43% of all patients) developed distant metastases, compared to 10% who developed locoregional recurrence, demonstrating the limitations of surgical resection, even in cases of R0 resection. The study also noted the role of adjuvant therapy to prevent this recurrence.

However, resection is limited by the ability to leave sufficient future liver remnant, along with the degree of compromise of the biliary tree and the possibility of biliary reconstruction [34]. Preoperative portal vein embolization should be performed in patients with low FLR to preserve volume of healthy liver post-surgery [35,36]. Portal vein embolization is also performed before hepatectomy for other liver cancers, such as HCC [37,38,39].

Alternatives to resection are not widely accepted for CCA. Liver transplantation has been performed for iCCA and is clearly not an option for most extrahepatic CCA. Studies have shown that in patients with small (<2 cm), “very early” iCCA, transplantation can lead to excellent 5-year survival (100%, 73%, and 73% at 1, 3, and 5 years) [40]. Another study sought to compare liver transplant to surgical resection with hepatectomy in patients with iCCA and hilar CCA. That study showed that liver transplant led to significantly improved survival (33% vs. 5%, *p* = 0.05) over the resection group [41]. They also included analysis of neoadjuvant and adjuvant therapies versus no additional therapies or simply adjuvant therapy, showing improved patient survival with the neoadjuvant and adjuvant therapies (47% vs. 20% vs. 33%, *p* = 0.03). Unlike other studies, they did not find that large tumor sizes were an independent predictor of poor outcomes. However, low survival rates in liver transplantation, with the added burden of the transplantation, make transplantation a poor option in many centers, though that has been ameliorated by newer development of guidelines for careful criteria [42,43]. These studies show the utility of transplantation in the surgery-systemic therapy-locoregional therapy paradigm.

In addition to surgical methods, pharmacologic treatments have been explored [44]. Early studies of chemotherapy-based approaches in CCA used gemcitabine and cisplatin, with the combination therapy superior to gemcitabine alone, with higher rates of tumor control and greater overall survival than gemcitabine alone (11.7 months vs. 8.1 months, *p* < 0.001) [45]. Current chemotherapeutic standard of care for CCA consists of gemcitabine and cisplatin. When used as an adjuvant, gemcitabine plus oxaliplatin has not been shown to improve survival or quality of life [46]. However, current standard of care for adjuvant chemotherapy for CCA is capecitabine [47]. This was based on the BILCAP trial, which compared capecitabine vs. observation following surgery, and showed superior overall survival [48].

Newer chemotherapies using immune checkpoint inhibitors have begun to be developed, but are currently second- or third-line behind gemcitabine and cisplatin [49]. PD-1, PD-L1, and CTLA-4 are immune checkpoints that have been targeted for CCA treatment and are expressed by activated T cells. Fibroblast growth factor antagonists have been introduced for CCA treatment, with clinical trials ongoing to optimize their use in the clinical setting, with several new drugs (derazantinib, infigratinib, erdafitinib, pemigatinib, and fuinatinib) undergoing clinical trials [50]. These treatments work on the finding that 10–15% of iCCAs have FGFR2 fusions, compared to eCCA, which very rarely present with FGFR alterations. Isocitrate dehydrogenase 1 and 2 have also emerged as a target, with clinical trials evaluating its use [51].

The lack of a comprehensive in vitro model of CCA has limited our ability to study and develop new treatments, partly because the in vitro model is limited in its two-dimensional structure, which does not represent the tumor environment [52]. Organoid-based model and in vivo models are also being investigated [44].

## 2. Locoregional Therapies in Cholangiocarcinoma

Like HCC, locoregional and interventional therapies have utility in unresectable cases of CCA. The liver receives 80% of its blood supply from the portal vein, compared to approximately 20% of its blood supply coming from the hepatic artery [53]. This division in blood supply has long served as an important anatomical framework for vascularly directed therapy in HCC [54,55,56]. In contrast to HCC, CCA is a less vascular tumor, suggesting a lessened role for these therapies. However, the data from many studies show survival benefit of interventional therapies with a vascular approach in cases of unresectable CCA [57,58,59,60]. These treatments are generally used for palliation in unresectable CCA, though disease control can also be achieved. Difficulties in studying these methods include the relative rarity of CCA, along with the subset of CCA patients who are not eligible for resection, creating a relatively small sample of patients for whom each non-curative treatment method is appropriate. Treatment response in patients receiving these therapies is generally assessed with the RECIST criteria [61,62]. An overview of the most common locoregional treatments in CCA is found in Table 1.

### 2.1. Transarterial Chemoembolization

The key principle behind transarterial chemoembolization (TACE) is that tumors require greater blood supply to support their increased metabolism compared to non-malignant tissue. To support this metabolic demand, the tumor promotes angiogenesis, creating blood flow from the hepatic artery. This creates a vulnerability, which multiple catheter-directed therapies exploit, to directly target tumor over other, healthy liver tissue. TACE is one such catheter-directed therapy that uses the approach by introducing chemoembolic agents that embolize the arteries supplying the tumor, leading to tumor cell death via cellular membrane disruption due to the induced ischemic state formed by the embolus [63]. Due to the anatomical approach of TACE, it is more commonly, and better, studied in iCCA than eCCA.

There are two primary variants of TACE in treatment use today: conventional TACE (cTACE) and drug-eluting bead TACE (DEB-TACE). In cTACE, chemotherapeutic agents are directly administered via the hepatic artery and its branches. By emulsifying the chemotherapeutic agent in lipiodol oil, the treatment effectiveness is increased with decreased washout, achieving higher concentrations and activity in the target tissue. DEB-TACE utilizes drug-eluting beads to target its agents to the tumor tissue. Both approaches allow superior targeting than systemic chemotherapeutic agents [57]. Broadly speaking, these two methods are similar in patient characteristics, OS, and response [64]. The role of TACE is generally as a replacement for surgical resection in patients with unresectable disease. One study showed that in patients with lymph node-positive disease or a positive resection, surgery has no survival benefit over either TACE method [65].

#### 2.1.1. Outcomes in Transarterial Chemoembolization for Intrahepatic Cholangiocarcinoma

Early studies in TACE were generally retrospective. One such study compared cTACE vs. supportive care for 155 patients enrolled between January 1996 and April 2009 [60]. The findings strongly supported the role of TACE, showing significantly increased survival for patients receiving TACE (12.2 months) over patients receiving supportive treatment (3.3 months). While no patients showed complete response, 23% of patients showed partial response and 66% showed stable disease. There was a significant rate of both hematological toxicities (13%), such as thrombocytopenia and hemoglobinemia, and non-hematological toxicities (24%), such as bilirubinemia and decreased albumin.

TACE has also been evaluated with systemic chemotherapy as an adjuvant. One study, which evaluated cTACE with cisplatin, doxorubicin, and mitomycin-C, evaluated survival, median time to progression, and complications in these patients [66]. The median survival from first treatment was 15 months, with 1-, 3-, and 5- year survival of 61%, 27%, and 8%, respectively. In their group of 62 patients, there were five major complications: pulmonary edema, pulmonary infarct, severe postembolization syndrome (PES), hyperglycemia, and one patient with acute renal failure and dehydration. All five of these patients recovered and were discharged. These results are significantly better than external beam radiation or systemic therapies [4].

There have been multiple meta-analyses including TACE therapies [64,67]. While the specific increase in survival varies amongst studies, there is a consistent result of increased survival of 207 months in patients receiving TACE treatments compared to systemic therapies, along with improvements in complications and disease progression. The variation between studies is possibly due to inclusion criteria and tumor features. The primary outcomes from TACE are based on palliative goals, with improved survival the most likely outcome.

However, some studies have examined TACE as a downstaging tool. One early study, which included 17 patients with unresectable iCCA, successfully downstaged 2 patients to surgical resection, with 1 of those patients disease-free at the time of publication [68]. Another more recent study of 109 patients showed that 4 patients (3.8%) were able to undergo resection following TACE therapy, with the majority showing stable disease following treatment. Most studies of TACE efficacy do not specifically include reports of downstaging, some due to experimental design.

#### 2.1.2. Transarterial Chemoembolization for Extrahepatic Cholangiocarcinoma

The anatomical differences between eCCA and iCCA have limited the number of studies on catheter-directed therapy in eCCA, but there is evidence to support the use of TACE in patients with eCCA. One retrospective study evaluated the use of cTACE using gemcitabine and cisplatin in patients with hilar cholangiocarcinoma (a division of eCCA) when used alongside radiotherapy [69]. They also examined stent patency, with all patients having received biliary drainage tube placement with or without biliary stent implantation. They found survival of the dual treatment (TACE plus radiotherapy) was 20.0 months, compared to 10.5 months in the control group (untreated with TACE or radiotherapy, *p* < 0.05), along with increases in stent patency. These data suggest efficacy of TACE in eCCA that is potentially similar to cases of iCCA.

#### 2.1.3. Side Effects in Transarterial Chemoembolization

One of the key side effects of TACE is post-embolization syndrome (PES). It is associated with longer hospital stays and recurrent admissions following multiple catheter-directed therapies in the liver for intrinsic and extrinsic liver malignancies [70]. It is found in up to 90% of patients undergoing hepatic chemoembolization and is thought to be due to off-target embolization leading to destruction of arteries supplying the stomach, duodenum, gallbladder, skin, or diaphragm. In PES, embolization leads to cytolysis which can occur in healthy liver tissue [71]. It presents with abnormalities in liver function tests, fever, nausea, malaise, loss of appetite, and abdominal pain [72]. Currently, there is no data to suggest variations in complications or complication rate between cTACE and DEB-TACE.

### 2.2. Transarterial Radioembolization

Transarterial radioembolization (TARE) utilizes similar techniques to TACE: targeted embolization via the hepatic artery leading to local ischemia and death. It is also called selective internal radiotherapy (SIRT) and utilizes a radioisotope of yttrium (Y^90^), which is bound to microspheres for targeting, in contrast to the TACE approached of chemotherapeutic agents bound to the microspheres [73]. Over a two-week period, the Y^90^ radioisotopes undergo beta decay, irradiating the tumor [74]. The precise mechanism for cellular death in this treatment continues to be explored [75]. The general procedure is similar to TACE, being a catheter-directed therapy through the hepatic artery to the specific region containing the tumor. Unlike external beam radiation, Y^90^ therapy allows increased radiation dosages to be delivered, which leads to improved anti-tumor activity [76].

One important difference compared to TACE is the latency of the treatment effect, brought about by the necessary two-week period during which the radioisotopes are undergoing decay and radiating the tumor, compared to the various effects of chemotherapeutic agents, which begin functioning over a much shorter time period. Another downside of TARE is that off-target Y^90^ radioisotopes pose a greater risk of non-local complications than TACE. This concern can be mitigated through a planning arteriography procedure performed shortly before treatment. This procedure has multiple aims: first, to map the tumor’s vascular anatomy to guide the TARE procedure and, second, to identify any extrahepatic vessels that may lead to off-target Y^90^ deposition and irradiation. These extrahepatic vessels can be embolized [77]. The lung is of particular concern due to the nature of vascular return from the liver going directly into the right side of the heart, followed by the vascular bed of the lungs. If a significant portion of the radioisotopes are depositing in the lung, the lung may undergo fibrosis. The last benefit of planning arteriography is that it enables careful modulation of treatment dosage to personalize for a specific patient. This is not possible for non-radioembolization [78].

#### 2.2.1. Outcomes in Transarterial Radioembolization

In addition to the use of catheter-directed therapies for palliation, data supports the use of TARE for downstaging otherwise unresectable iCCA for later resection. One study followed 45 patients whose iCCA was judged to be unresectable, and each patient was treated with both chemotherapy (gemcitabine and/or platinum salts) and radioembolization [79]. Every two months, the patients were re-evaluated for tumor response and potential for resection, including any possible complete removal of the tumor, regardless of technique or margin width. Of the 45 patients, many were ruled out of resection for extrahepatic disease, multiple lesions, tumor recurrence, or cirrhotic disease precluding resection, but eight patients ultimately underwent surgical resection with curative intent. Two patients did not survive to discharge due to Clavien-Dindo complications of grade three or greater. Another patient died 6.5 months following surgery without evidence of disease recurrence. The remaining five patients were alive at time of publication, with two of those five experiencing recurrence. Median disease-free survival was 19.1 months, compared to survival in patients with unresectable disease of less than a year, depending on supportive care [80]. While the number of patients for which this treatment course was appropriate was low, with only 8 of the 45 unresectable iCCAs utilizing TARE for downstaging, TARE effectively extended survival and provided disease-free survival. One systematic review of TARE outcomes reviewed 12 studies using Y^90^-based treatments, included 298 patients [81]. Of these, 7 patients from 3 separate studies were successfully downstaged to surgery, supporting the role of TARE in downstaging, albeit for a small minority of patients. A more recent study that examined the response rate of patients with iCCA to TARE alongside systemic chemotherapy showed that 22% of patients were successfully downstaged to surgical resection, with 8 of those 9 patients achieving R0 margins from resection [82]. This study also found that the majority of patients successfully downstaged had tumor limited only 1 hemiliver.

The primary use of TARE in CCA is for palliation. One important study compared TACE and TARE treatments to uncover differences in survival and side effect profiles between these approaches, with the caveat that treatment selection between these two treatments is dependent on numerous factors, such as liver function, stage of disease, previous treatments, and comorbidities [64]. 31 studies were included in their meta-analysis, with 906 patients in their TACE group and 789 patients in the TARE group. Median survival across these groups were not significantly different (14.2 months vs. 13.5 months). These studies reflect the majority of the clinical utilization of TARE, as a palliative method to control local disease and prolong survival.

#### 2.2.2. Safety of Transarterial Radioembolization

The purpose of TARE is to provide higher, more targeted doses of radiation to the tumor than possible with external beam radiation. However, these doses are sufficient to cause significant damage to non-tumor tissues. Clinical toxicities associated with TARE include abdominal pain, nausea, vomiting, anorexia, albumin toxicity, and bilirubin toxicity [83]. Radiologic findings following TARE include ascites, pleural effusion, and pulmonary embolus [84]. Comparisons of rates of adverse events between TACE and TARE therapies for iCCA show lower rates of clinical adverse events in TARE treatments (43%) than TACE (58.5%), particularly in PES syndrome [64]. These findings support the superiority of TARE in patients who cannot tolerate or are less likely to tolerate TACE.

### 2.3. Percutaneous Ablation

In addition to embolization, multiple techniques have been used to ablate iCCA tumors. Like embolization, these techniques are generally limited to unresectable iCCA due to their inferiority to surgical resection and lack of efficacy in extra-hepatic disease [85]. Originally used primarily for palliation, multiple studies have shown the benefits of ablation in slowing tumor progression and improving survival.

The most commonly performed method of ablative therapy for iCCA is radiofrequency ablation (RFA), which uses alternating electrical current at a high frequency to heat tissue via rapid electron vibration. This heat directly causes cell death, leading to anti-tumor efficacy. The objective in RFA is to heat tissues to a50–100 °C for 4–6 min without the vaporization or charring that can occur at higher temperatures [86]. Efficacy of RFA is determined by whether this delivery of thermal energy sufficiently ablates the tumor.

Microwave ablation (MWA) is a technique similar to RFA that also utilizes thermoablative methods to damage tumor tissue. MWA uses an oscillating microwave field to induce cell death, with the advantage of a reduced heat sink effect in comparison to RFA. The heat sink effect occurs when adjacent vascular structures dampen the ablative effect of thermal energy by reducing the thermal differential between the target tissue and the rest of the body, in this case conducting thermal energy away from the target site.

Cryoablation works on a similar, inverse principle of cell death due to decreasing heat in the tumor, leading to cell membrane and organelle damage [87]. Ice crystal formation in the target site leads to osmotic pressure changes and dehydration [88]. There is very limited data, with no studies focusing on cryoablation for CCA, like there have been for HCC, for which cryoablation is a validated treatment method.

#### Outcomes in Percutaneous Ablation

There have been multiple reviews on the efficacy of thermo-ablative techniques in the context of iCCA [85,89]. These studies have found that the RFA is significantly more commonly performed, with 83.7% of patients undergoing RFA vs. the remaining 16.3% undergoing MWA. Most of these procedures were performed percutaneously, with a small minority (only 4 patients) undergoing open RFA. In the available studies, they found overall survival ranging widely, from 8.7 months to 52.4 months, indicating significant disparity between centers, and survival measures varied in method between studies. The 5-year median survival ranged from 15–83.3%, which represents an improvement over surgical resection. Pooled analysis of 5-year survival was lower, at 16%. This suggests that for patients for which ablation is possible, ablation may be an acceptable primary treatment option, though this study is not appropriate for independently making a broad recommendation overturning surgical resection as the gold standard of iCCA treatment.

Outside of survival measures, percutaneous ablation performs quite well. It has a lower risk of complications and does not independently require inpatient admission, compared to resection. Overall, it reduces hospital admission [90]. However, there are multiple complications reported: hemorrhage, infection, tumor seeding, thermal injury, and incomplete tumor ablation can occur following RFA [91]. Despite the documented advantages of MWA in that it is less susceptible to the heat sink effect and has faster heat generation, there is no evidence that MWA has a lower rate of complications [92]. This represents a potential for further studies to investigate, though running trials directly comparing these relatively rare treatments may make recruitment difficult.

## 3. Other Therapies

Histotripsy is another locoregional therapy that utilizes ultrasound as an ablative tool, in contrast to thermoablative methods. It is noninvasive and nonionizing and has been performed in treating multiple liver tumors. Recently, studies have begun to investigate the feasibility of histotripsy to target CCA [93]. In addition to the improvements in complications brought on by the non-invasive nature of histotripsy, there is evidence to suggest that it can be applied to eCCA because it is not limited by the same vascular access as iCCA. Nonetheless, current published studies on histotripsy for CCA are on in vivo mouse models and ex vivo liver tumor specimens, highlighting the gap between current practice and implementation for human treatment.

In addition, several more technologies have been explored for CCA. Proton beam therapy has been used to irradiate tumors, data showing patients being treated with the goals of both cure and improved survival [94,95]. Irreversible electroporation is an ablative technique that uses electrical voltage instead of thermal energy. Data is limited on irreversible electroporation, with some evidence of reducing disease burden [96].

## 4. Discussion

Locoregional therapies, in the context of CCA, provide three primary roles: (1) downstaging to surgery, (2) palliation (by achieving local control), and (3) cure. In each of these roles, the first-line treatment for CCA is surgical resection of the tumor, but numerous anatomical and clinical parameters make this impossible in a large number of patients, leading to use of locoregional therapy with these goals. Of these three roles, cure is rare and is generally not the goal of locoregional therapies. Downstaging is most apparent in the case of TARE and TACE, with limited information on its role in ablation-based methods.

TACE, TARE, and ablation are the most commonly performed locoregional therapies for treatment of CCA. As all are locoregional therapies directed at the liver, they have limited use for extrahepatic disease and there is limited data on eCCA, though all three have been studied extensively in iCCA. TACE is the most common, with significant data showing improvements in survival for patients, but TARE is shown to have improvements in side effects, with comparable improvements in survival. However, ablation has the strongest evidence to suggest a role beyond palliation, with effective downstaging bridging patients to surgical resection, the treatment with the best efficacy for cure. Other treatments are being developed, largely based on similar treatments for other liver malignancies, primarily HCC, but the data is extremely limited. One of the most significant obstacles in the development of locoregional treatments for CCA is the relative rarity of CCA combined with the superiority of surgical resection for all appropriate patients, which complicates patient recruitment and limits the power of potential studies.

## 5. Conclusions

CCA is a deadly disease with a poor prognosis, due mainly to the lack of curative treatments outside surgical resection. The advent of locoregional therapies allow superior palliative care than previous systemic therapies with improvements in both survival and side effect profile. New data is beginning to show roles for locoregional therapies outside mere palliation. The opportunity for downstaging to surgical resection further broadens the opportunities to utilize locoregional therapies. Further exploration into these therapies is still necessary, particularly in the realm of extrahepatic CCA, but the data suggests that locoregional therapies will continue to play an important role in the treatment of CCA for the foreseeable future.

## Figures and Tables

**Table 1:** Overview of current locoregional therapies.

Treatment	Technique	Roles in Treatment	Limitations	Safety Profile
Transarterial Chemoembolization (TACE)	Lipiodolized chemotherapeutics or drug-eluting beads embolize tumor-supplying arteries	Palliation in unresectable CCA, and downstaging	Toxicity, choice of agent	PES, fever, nausea, malaise
Transarterial Radioembolization (TARE)	Yttrium-90 internally radiates tumor tissue	Palliation and downstaging	Pre-procedure angiography, iCCA only	PES, improvement over TARE
Ablation	Thermal energy directly damages tumor tissue	Palliation and disease control	Heat sink effect, iCCA only	Does not require inpatient admission

## Data Availability

Not applicable.
